# Temperature and soil nutrients drive seed traits variation in *Pterocarpus erinaceus* (African rosewood) in Ghana

**DOI:** 10.1002/pei3.10120

**Published:** 2023-07-25

**Authors:** Padmore B. Ansah, Shalom D. Addo‐Danso, Ebenezer J. D. Belford, Joseph M. Asomaning, Abena B. Asare‐Ansah, Naomi A. Fosu, Rosalinda A. Ankobiah

**Affiliations:** ^1^ Department of Theoretical and Applied Biology, Faculty of Biosciences, College of Science Kwame Nkrumah University of Science and Technology Kumasi Ghana; ^2^ CSIR‐Forestry Research Institute of Ghana Kumasi Ghana; ^3^ Department of Urban Forestry and Natural Resources Southern University and A&M College Baton Rouge Louisiana USA; ^4^ CSIR‐Crops Research Institute Fumesua Ghana

**Keywords:** germination, maternal environment, phenology, provenance, *Pterocarpus erinaceus*, seed traits

## Abstract

Among plant populations, variation in seed traits has important consequences on species recruitment and performance under different environmental conditions. Knowing such variations and understanding its environmental drivers could help with conservation efforts that protect against the loss of diversity. This information is however lacking in the extinction‐threatened *Pterocarpus erinaceus* Poir (African rosewood) in Ghana. Here, we assessed variation in seed set, seed morphological and chemical traits, germinability, and seedling growth of African rosewood from four distant provenances (Tumu, Wa, Carpenter, and Ejura) in Ghana. We sought to answer how local environmental conditions influence the expression of seed traits by examining the relationship between seed traits and maternal environmental factors (temperature, rainfall, soil nutrient, and vegetation index) using regression models and correlation analysis. Seed size, mass, and nutrient composition differed considerably among provenances. Seed size and mass increased as the seed source moved further away from the forest‐savanna transition toward the Guineo‐Sudanian savanna regions. Temperature mainly accounted for the variability observed in seed traits. Phenology curves of the seed source vegetation expressed a strong correlation with monthly rainfall. Overall, the occurrence of samara containing whole seeds was low (<50%) except for Tumu provenance. Seeds were rich in carbohydrate and crude protein content consistent with most leguminous plants while mean percentage germination ranged between 30 and 62% among provenances. Our results highlight the adaptive strategies of African rosewood to different environments through the expression of their seed traits and suggest the need for priority action to maintain its conservation.

## Graphical abstract

Seed germination performance of Pterocarpus erinaceus provenances demonstrates adaptive responses that are reflective of their maternal environment. The highest mean percentage of seed germination was realized at 62%.

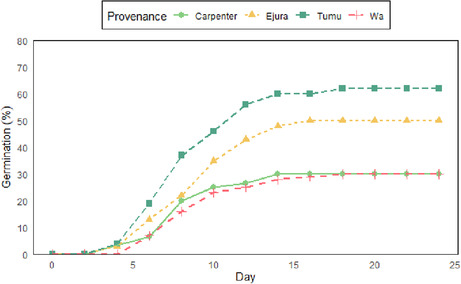

## 1 INTRODUCTION

Seed production and seed traits in flowering plants are important aspects of their life history with direct influence on the plants' fitness and persistence. Seed traits associated with recruitment may influence species resilience and ultimately reduce the risk of extinction against disruptions from the rapidly changing climate (Carón et al., 2014; Cochrane et al., 2015; Wu et al., 2018). Higher seed production may as well increase the proportion of seeds that escape predation and transition onto seedlings thereby contributing to plant persistence (Perea et al., 2013). Both seed production and seed traits are controlled by genetic and environmental factors (Bradford & Nonogaki, 2008; Cochrane et al., 2015). The genetic factors originate from genes of the parental plants fusing together and expressed in the embryo and endosperm during seed development (Bradford & Nonogaki, 2008).

Environmental factors refer to external factors that affect the maternal plant during seed development. Researchers typically refer to these environmental effects as the influence of the “maternal environment” and specifically define maternal environment as the environmental effects on developing seeds, with the direct involvement of tissues of the mother plant and mediated by the genome or epigenome of the mother plant (Penfield & MacGregor, 2017). Empirical evidence in different plant species has confirmed strong maternal environmental effects on the phenotype and fitness of offspring (e.g., Galloway, 2005; Galloway & Etterson, 2007; Herman & Sultan, 2011; Penfield & MacGregor, 2017). Again, increasing evidence suggests that these environmental effects are adaptive and transgenerational enhancing the capacity of offspring to deal with stress (Galloway & Etterson, 2007; Herman & Sultan, 2011; Zas et al., 2013).

Seed provisioning is one of the most important transmission vehicles of maternal environmental effects (Zas et al., 2013). It refers to the hormones, transcripts, and nutrient reserves (carbohydrate, protein, lipids, etc.) allocated to developing seeds by the maternal plant (Donohue, 2009; Galloway, 2001; Herman & Sultan, 2011). Within species, seed provisioning is environment dependent such that limited light, water, and nutrient resources during seed production result in reduced sizes, growth rates, and competitive capacities of seeds and seedlings (Fenner & Thompson, 2005). For instance, the size of seeds could be a reflection of stored nutrient reserves, while the seed nutrient may also reflect soil nutrient availability to the mother plant (De Frenne et al., 2011; Pérez‐Ramos et al., 2010). However, several studies have reported the production of bigger seed sizes and mass (i.e., increased provisioning by maternal plants) but often with fewer seed sets in certain species growing in resource‐limited environments, which could be an adaptive response to maximize seedling survival (Herman & Sultan, 2011). Thus, maternal seed provisioning and by extension maternal environmental effects are major drivers of seed traits and subsequent seedling establishment, while the direction of effect is species‐dependent.

Besides the abiotic maternal environmental effects, herbivory, predation, and pathogen infection of maternal plants are known to influence seed production and seed traits (Herman & Sultan, 2011). In some wind‐dispersed tree genera, *Ulmus*, *Salix*, and *Pinus*, trees with seed predation history tend to develop high levels of empty fruits (samara) and undeveloped seeds. The phenomenon is explained to have ecological importance for the trees, being an adaptive mechanism to discourage seed predators and increase the proportion of full and viable seeds that escape predation both before and after dispersal (Perea et al., 2013; Zangerl et al., 1991).

Despite the importance of environmental controls for species conservation and management, variations in seed traits and development and its environmental drivers in *Pterocarpus erinaceus* remain poorly explored. It is also not known how seed trait variations in African rosewood influence germination and seedlings growth. Again, reports on the germination capacity of African rosewood in literature are divergent. For instance, Amponsah et al. (2022), Duvall (2008), and Tiika et al. (2019) have reported high emergence of untreated seeds (above 70%) and pre‐treated seeds (between 70% and 100% with different pre‐treatment methods) in Ghana. On the contrary, experiments conducted in Burkina Faso and Ghana reported emergence of 70% and 50%, respectively (Kyei, 2016; Zida et al., 2005), while low germination of less than 50% in a separate study conducted in Benin have been reported with pre‐treated and untreated seeds (Akpona et al., 2017). It is unclear for us if variation in seed traits and germination capacity in African rosewood is a result of the geographical origin of the seeds, and hence differences in maternal environmental effects during seed production or some other factor. Addressing this knowledge gap will improve the cultivation and conservation success of the species.

Here, we compared seed morphological and nutrient composition characteristics of African rosewood from four provenances in Ghana and associated them with the maternal environment. Again, we assessed seed germination performance from each provenance and related them with the seed nutrients and the maternal environment. We conducted a 6‐month seedlings growth experiment to compare provenance performance in a predetermined optimum growth environment of the species. We hypothesized that (1a) maternal environments (provenances) with limited resources and/or with pronounced environmental stress will influence the adjustment of seed morphological traits to have a better competitive advantage (i.e., larger sizes and bigger mass) and that (1b), producing seeds of larger sizes and bigger mass will maximize germination success. Larger seed sizes and masses are known to exhibit greater tolerance to heat and other environmental stresses, with higher seed germination and growth rates. Therefore, in limited resources and stressed environments also associated with higher metabolic costs, we expected mother plants to invest more in size and mass of seeds (Calvo et al., 2016; Murray et al., 2004; Pérez‐Ramos et al., 2010). (2a) Seed nutrient composition will reflect the soil nutrient characteristics of the maternal environment and (2b) will affect the germination outcome of the species. It is well established that maternal seed provisioning represents a potential mechanism for observed variations in nutrient composition of seeds of the same species, as also germination and early establishment strongly depend on seed nutrient reserves (De Frenne et al., 2011; Pérez‐Ramos et al., 2010). (3) Provenances with larger seeds and bigger seed mass will have a better seedling growth performance. Since seedlings of larger seeds are known to have higher survival through time, we expected provenances with larger seeds to produce seedlings of vigorous growth characteristics that enhance survival and establishment (Moles & Westoby, 2006).

## 2 MATERIALS AND METHODS

### 2.1 Description of species

*Pterocarpus erinaceus* Poir (African rosewood) is a medium‐sized leguminous tree species (Fabaceae, subfamily Papilionoideae) endemic to the dry forests and savanna woodlands of West Africa (Arbonnier, 2002). The species, which attains a height ranging from 12 m to 15 m and a girth of 1.2 m−1.8 m at maturity, is heavily exploited for its timber in international trade (Dumenu, 2019). It is estimated as the most traded tropical hardwood worldwide (Lawson, 2015). Locally, the leaves serve as animal fodder, the stem bark and root have several ethnobotanical uses, and the wood is preferred for burning charcoal and the production of musical instruments (Duvall, 2008; Korang et al., 2015; Ouédraogo et al., 2006). The species has a wide distribution with an estimated extent of occurrence exceeding 2 million km^2^. This wide geographical range is expected to confer substantial intraspecific variability that improves the species’ performance under varying environmental conditions (Adjonou et al., 2020; Duvall, 2008; Messier et al., 2010; Segla et al., 2016).

### 2.2 Study provenances, collection, and extraction of seeds

**FIGURE 1:**
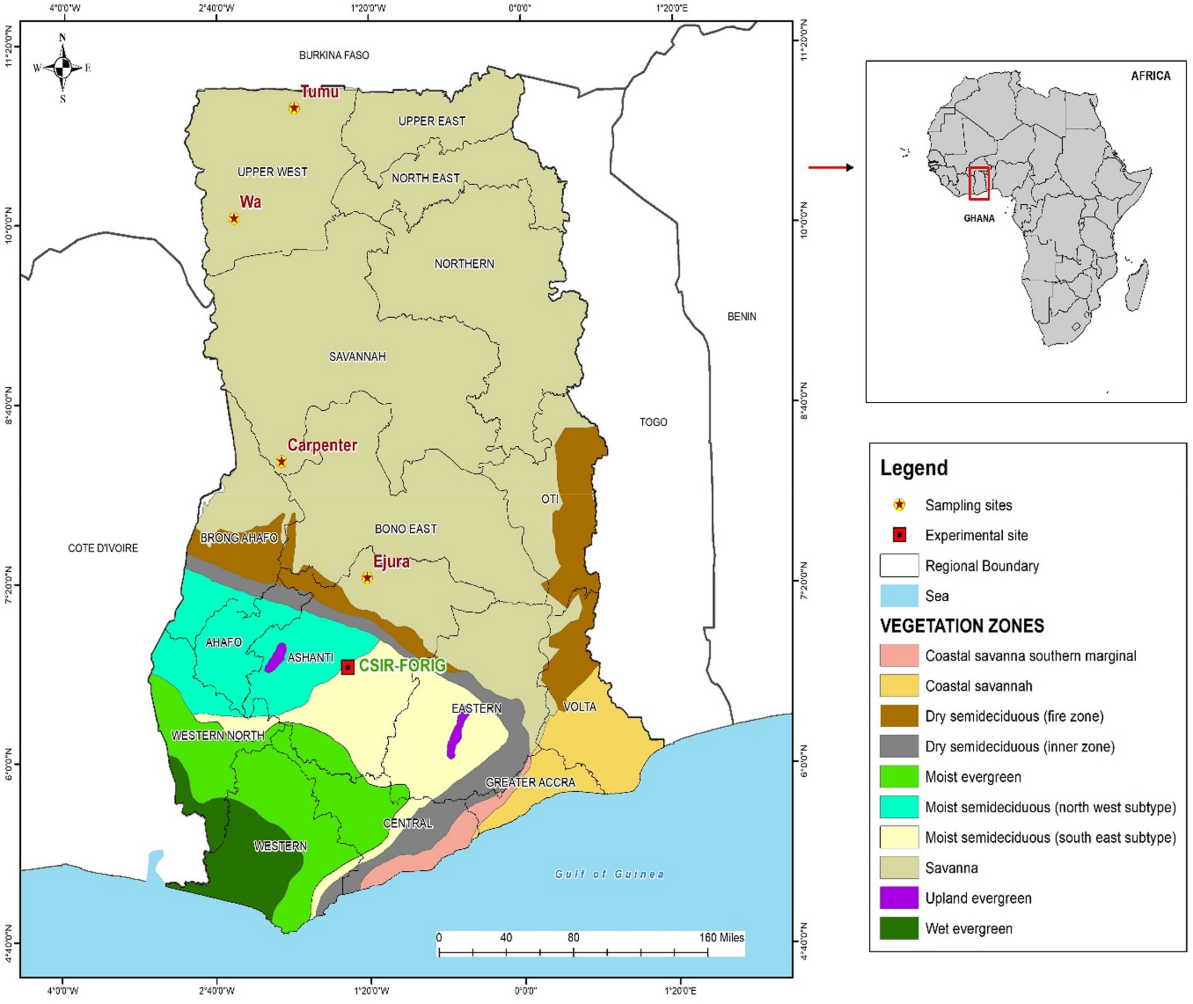
Location of study provenances of *Pterocarpus erinaceus* across its geographical distribution in Ghana.

Four African rosewood populations (hereafter referred to as provenances) spatially separated within the savanna zones in Ghana were selected for this study (Figure 1). They are the Ejura, Carpenter, Wa, and Tumu provenances with GPS coordinates ranging between latitude 7°23'N to 10°57'N and longitude 1°21'W to 1°59'W, respectively. Ejura occurs in the forest‐savanna transition zone while Carpenter, Wa, and Tumu occur in the Guinea savanna ecological zone. These provenances were selected because of the occurrence of high density of African rosewood trees, and their importance in supplying seeds for various afforestation progras in the country. The large distance between these provenances (approximately 526 km between the two farthest provenances) also allowed for examining possible variations among African rosewood populations in Ghana. Matured fruits (Samara) at the point of natural abscission were harvested from plus‐trees (*N* = 3–5) in each provenance in February. Plus trees are phenotypically superior trees and form the basis of efficient and reliable seed collection (Jo & Wilson, 2005). In Ejura and Carpenter, fruits were ready for collection during the first week of February, while in Wa and Tumu fruits matured in the last week of the same month. The selected plus‐trees were separated from each other by a minimum of 200 m distance to reduce the chances of harvesting seeds from genetically closely related mother trees. A maximum of 20% of a tree's crown was removed during seed harvest according to Royal Botanic Garden, Kew recommendations (Royal Botanic Gardens, Kew, 2005). This was to ensure that sufficient seeds were harvested while the mother trees were not endangered by the act of harvesting. Seeds were extracted from the samara by cutting the thorny winged pericarp with a pair of scissors without inflicting damage on the seeds.

### 2.3 Environmental characteristics

Monthly rainfall and temperature records were obtained for each provenance location in the seed production year (March, 2019 to February, 2020), which covered the season of leaf flushing and subsequent growth, flowering, fruiting, and seed development up to the time of fruit harvest. Rainfall data were obtained from the Ghana Meteorological Agency (GMA) except for Tumu, where data were obtained from the office of Ministry of Food and Agriculture at the Sissala East Municipality. The rainfall pattern in each provenance location was characterized by determining the seasonality index of rainfall over the seed production year. Seasonality index ($\bar{\mathrm{SI}}$) proposed by Walsh and Lawler (1981), is defined as the sum of absolute deviation of mean monthly rainfall from the overall monthly mean divided by the mean annual rainfall (Livada & Asimakopoulos, 2005). It is calculated using the equation (Walsh & Lawler, 1981):

$$\bar{\mathrm{SI}}=\frac{1}{\bar{R}}\sum_{n=1}^{n=12}\left|\bar{x_{n}}-\frac{\bar{R}}{12}\right|$$

Where $x_{n}$ indicates the mean rainfall of month *n* and $\bar{R}$ is the mean annual rainfall.

The index varies between zero (where the amount of rainfall is equal for all months in the year) and 1.83 (when all rainfall occurs in a single month). We chose this parameter ($\bar{\mathrm{SI}}$) because it allows for the quantification of the seasonal variations in rainfall and hence spatial comparisons of rainfall seasonality. Again, the values are easily interpretable by comparing them with a standardized range of values and their explanations.

Temperature data for Wa and Carpenter were obtained from the GMA, while Tumu and Ejura records were sourced from the Moderate Resolution Imaging Spectroradiometer (MODIS) Terra Land Surface Temperature and Emissivity Daily Global datasets at 1 km spatial resolution on Google Earth Engine (GEE) data catalog. GEE is a cloud‐computing platform that houses satellite images with different temporal and spatial resolutions on a global scale (Gorelick et al., 2017). We resorted to MODIS for temperature data of Tumu and Ejura provenances because they were not available from the GMA weather stations. However, MODIS Land Surface Temperature has been found reliable, agreeing well with air temperature measurements of weather stations (Hachem et al., 2012). The mean monthly temperature over the seed production year was calculated and subsequently used in the regression models.

Soil was sampled to 10 cm soil depth at four randomly located points at the base of each plus tree where the fruits were collected (McCarthy, 1997). Soil samples were placed into labeled plastic bags, and transported to the laboratory at the CSIR‐FORIG for further processing. At the laboratory, soils were thoroughly mixed to obtain a representative composite sample per provenance, and afterward were air dried, ground, and sieved, and subsequently sent to the CSIR‐Soil Research Institute (CSIR‐SRI) for chemical and physical analyses. At CSIR‐SRI, the soils were analyzed for concentrations of soil organic matter (SOM), soil organic carbon (SOC), pH, total nitrogen (N), available phosphorus (P), exchangeable potassium (K), sodium (Na), magnesium, (Mg), calcium (Ca), base saturation, total exchangeable bases, exchangeable acidity, effective cation exchange capacity (E.C.E.C), and particle size composition following standard procedures as described in McCarthy, (1997). SOM was determined by the wet digestion method and SOC was analyzed by titration following digestion of soil samples in K_2_Cr_2_O_7_‐H_2_SO_4_ solution on a heating panel. Available nitrogen was analyzed by the modified Macro‐Kjeldahl method. Available phosphorus was extracted with Bray's P solution and measured on a spectrophotometer. Sodium and potassium contents were determined by flame photometry, while calcium and magnesium were determined by atomic absorption spectrophotometry. Exchangeable bases were extracted with 1.0 M ammonium acetate solution, and pH was determined by a pH meter in 1:2.5 soil: water suspension. Effective cation exchange capacity (ECEC) was calculated as the sum of exchangeable cations (K, Ca, Mg, and Na) and exchangeable acidity (Al + H). Particle size analysis was done using the pipette method.

Normalized difference vegetation index (NDVI) is a vegetation index that correlates strongly with aboveground net primary productivity (Pettorelli et al., 2005). It is used to assess whether the target observed contains live green vegetation (chlorophyll) or not. NDVI is a commonly used index in assessing vegetation health (Mkhabela et al., 2011). It has the ability to predict land cover changes, plant seasonal cycles, and crop yield (Meneses‐Tovar, 2011; Pettorelli et al., 2005). It is calculated using the formula:

$$\text{NDVI}=\;\frac{\left(\mathrm{NIR}-\mathrm{Red}\right)}{\left(\mathrm{NIR}+\mathrm{Red}\right)}$$

Where NIR (near infrared) and red lights are reflected bands of wavelengths of the sunlight. It uses the underlying principle that for vegetated surfaces, red wavelengths are characterized by high absorption by leaf chlorophyll and hence low reflectance, while near‐infrared (NIR) wavelengths are characterized by low absorption and hence high reflectance. Where vegetation is under stress, the reflectance values change in the opposite direction (Mkhabela et al., 2011). NDVI values are unit‐less and range from −1 to +1 where negative values represent areas without vegetation, that is, open water, snow, ice, or clouds. Rocks and bare soils have values closer to zero, while values above 0.2 indicate plant activity (Mkhabela et al., 2011; Pettorelli et al., 2005). Composite images downloaded from Landsat 8 over the seed production year with a 30 m spatial resolution were analyzed eliminating those contaminated with clouds (Meneses‐Tovar, 2011). A total of 146, 74, 146, and 73 composite images between March 1, 2019 and February 29, 2020 were obtained for Tumu, Wa, Carpenter, and Ejura provenances, respectively, and processed with Google Earth Engine. NDVI was calculated for all the images and used to generate phenology curves of the seed source vegetation over the period. The mean NDVI of all images for each provenance was calculated and used in the regression models.

### 2.4 Seed characteristics

Seed set was observed from randomly selected 300 fruits from each provenance and scored for the occurrence of whole seeds, underdeveloped seeds, and empty‐seeded samaras. The term underdeveloped used here refers to an aggregation of features which include partially formed seeds, aborted seeds, shrunken seeds, and predated seeds (Perea et al., 2013; TeKrony & Hardin, 1969). Seed mass was determined for each provenance by counting 8 replicates of 100 whole uncut seed samples and weighed with an electronic balance. The average weight of the replicates was then determined. Ten (10) whole seeds of each provenance were selected, scanned with an HP scanner (CanonScan LiDE 220), and the images imported into the pixel counting software, Image J for size (area and length) examination (Schneider et al., 2012). Freshly extracted seed samples from each provenance were ground and analyzed for nutrient composition following standard protocols described by the Association of Official Analytical Chemists (Association of Official Analytical Chemist, 2002) at the Laboratory of Food Science and Biochemistry, Kwame Nkrumah University of Science and Technology. The components analyzed were moisture, ash, fiber, protein, fat, carbohydrate, and nitrogen.

### 2.5 Germination and seedlings growth

Germination and growth experiment of seeds and seedlings from all provenances were conducted at a shade house with approximately 15.6% irradiance of unshaded sunlight situated at the plant nursery of the National Tree Seed Center (NTSC), CSIR‐Forestry Research Institute of Ghana (CSIR‐FORIG). A mean photosynthetic photon flux density (PPFD) of 73.5 μmol m^−2^ s^1^ at the shade house was recorded over the period using quantum sensors (±5% accuracy, Model MQ‐200, Apogee Instruments). In the germination experiment, 5 replicates of 20 whole seeds each were sown in germination bowls filled with river sand for each provenance except Carpenter where we used three replicates as a result of inadequate seeds. The set‐up was watered every morning and germination was observed and scored up to 24 days after sowing by which time germination had ceased (Poorter, 1999). Successful seedlings were subsequently transplanted into medium‐sized polypots (13 cm × 18 cm) for the growth experiment. A mixture of 2:1 river sand and forest soils was used as growth media for the potted seedlings (Poorter, 1999). The predominantly sandy soil was important to simulate the texture of the natural soils in rosewood growing areas as *P. erinaceus* naturally occurs in poor loose soils (Barstow, 2018). Again, the texture improved drainage with the regular watering of seedlings while the forest soils served as nutrient base (Amissah et al., 2015). The seedlings growth was observed for 6 months during which the stem length, root length, and root collar diameter growth were measured. Relative growth rates of the stem length and root collar diameter were calculated for each provenance using the 1st‐ and 6th‐month seedlings growth measurements with the equation (Hoffmann & Poorter, 2002):

$$\mathrm{RGR}=\left(\frac{\bar{\ln X_{2}\;}-\bar{\ln X_{1}}}{∆t}\right)$$

Where $X_{2}$ and $X_{1}$ are the variables measured in the two assessments (6th and 1st month, respectively). ∆t is the time interval between the two assessment occasions in weeks.

### 2.6 Statistical analysis

All data sets were tested for assumptions of normality and homogeneity of variances using Shapiro–Wilk's test and Levene's test, respectively. Seed morphological traits (area, length, and mass) were compared among provenances using Kruskal‐Wallis rank‐sum test because of unequal variances and non‐normality of the data. Subsequent pairwise comparisons were conducted using Dunn's test with a 95% confidence interval. Chi‐squared test was used to compare the number of counts in each seed set characteristic (i.e., whole, underdeveloped, and empty‐seeded samara) among provenances. The seedlings growth measurements were log‐transformed to meet assumptions of normality before subjecting the data to a one‐way analysis of variance.

We investigated germination response to the maternal environment and its proximate composition using generalized linear models (GLMs). The GLMs were adopted because of their suitability in analyzing non‐normal heteroskedastic data characteristic of binomial germination proportions (Gianinetti, 2020). The models were fitted using the glm function in the “lme4” package in R statistical software. Germination time curves for the provenances were derived using functions in the “GerminaR” package in R statistical software.

To investigate the effects of soil physicochemical characteristics on seed traits, a dimensionality reduction analysis was conducted to reduce the dimensions of the soil variables into principal components. The first and second PCA axes were extracted which accounted for 47.2% and 30.8% of the variability in the soil parameters, respectively. The first axis correlated with the majority of the soil nutrients, mainly organic carbon, total nitrogen, organic matter, Mg, T.E.B, E.C.E.C, Silt, and clay content (*r* > 0.73). The second axis correlated with potassium and exchangeable acidity (*r* > 0.87, Supplementary Table S2).

We studied the relationship between the maternal environment and seed morphological traits (i.e., seed area, seed length, and seed mass) using a linear mixed‐effects model. The fixed effects were mean monthly temperature, absolute rainfall (i.e., total rainfall amount received during the seed production year), the two soil PCA axes, and NDVI, while provenance locations were included as random effects. All values of the explanatory variables were scaled or standardized (i.e., by subtracting the mean value of the variable and dividing by the standard deviation) to facilitate comparisons among variables before being included in the model. After the initial model fit with all potential variables, a model simplification procedure was carried out by dropping non‐significant explanatory variables, one per time to select the model only retaining variables that collectively resulted in the lowest value of the Akaike Information Criterion (AIC) (Wu et al., 2018). The models were fitted using functions in the “lme4” and “lmerTest” package in R statistical program version 4.2.3 (R Development Core Team, 2015).

## 3 RESULTS

### 3.1 Variation in the seed production environment among provenances

Rainfall occurrence was generally seasonal and differed among provenances. Total rainfall recorded were 905 mm, 1133 mm, 1168 mm, and 1244 mm for Carpenter, Wa, Ejura, and Tumu, respectively. The rainfall pattern in Ejura was bimodal while all others showed a unimodal pattern (Supplementary Figure S1). Carpenter had the highest seasonality index of rainfall and was similar for Wa and Tumu (0.97, 0.94, and 0.93, respectively). Seasonal patterns in rainfall in these three places are classified as “Markedly seasonal with a long drier season” based on Walsh and Lawler (1981). Rainfall in Ejura was less variable, differed from the three with a seasonality index of 0.76, and falls in the “Seasonal” category.

The soils were predominantly sandy with sand, sandy loam, sand clay loam, and sand texture classifications for Tumu, Wa, Carpenter, and Ejura, respectively. Soils were slightly acidic across all provenances and differed in their nutrient‐supplying capacity, especially for Mg, P, and organic matter (Supplementary Table S1). Clay and silt content were relatively high for soils from Wa and Carpenter provenances.

**FIGURE 2:**
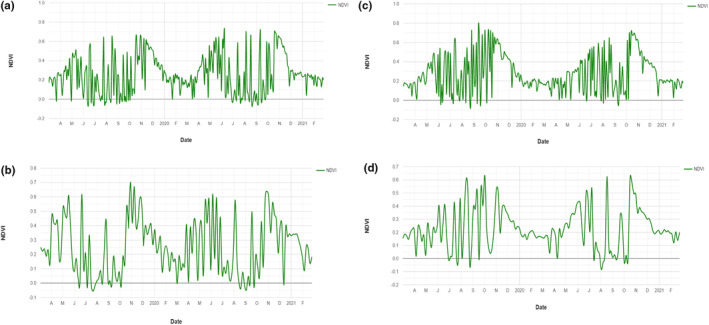
(a) Normalized difference vegetation index (NDVI) time curve describing phenology of the seed source vegetation of the Carpenter provenance of *Pterocarpus erinaceus* over the study period. (b) Normalized difference vegetation index (NDVI) time curve describing phenology of the seed source vegetation of the Ejura provenance of *Pterocarpus erinaceus* over the study period. (c) Normalized difference vegetation index (NDVI) time curve describing phenology of the seed source vegetation of the Tumu provenance of *Pterocarpus erinaceus* over the study period. (d) Normalized difference vegetation index (NDVI) time curve describing phenology of the seed source vegetation of the Wa provenance of *Pterocarpus erinaceus* over the study period.

The phenology curves (Figure 2a–d) highlight the growth patterns of the seed source vegetation in each provenance over the seed production year as well as the subsequent year. The subsequent year's growth was included to show the recurring seasonal growth cycles of the seed source stands. The phenology for all provenances displayed strong synchrony with the monthly rainfall pattern recorded over the same period (see rainfall pattern in Supplementary Figure S1). The trajectory of the phenology curves displays a rise in NDVI values (values increase from 0.2) in April and a peak in October, which is in conformity to the onset of rains in March and April. Declines in reflectance values at different points and the sinusoidal trends reflect the seasonal dynamics of the African rosewood predominant vegetation of the seed sources. It should be noted that the NDVI values reported are the combined response of the whole vegetation (grasses, shrubs, and other tree layers) of the seed source. In Ejura, the curve peaks at two separate times of the year, that is, May/June and October/November with a long interval decline (Figure 2b). This mirrors the bimodal rainfall pattern and the high monthly rainfall values recorded in these months in Ejura. In Carpenter, the NDVI increased from April through May, then displayed intermittent dips between June and September until it finally peaks in October (Figure 2a), which mirrors rainfall patterns across months during the year in this provenance.

In Tumu and Wa (Figure 2c,d, respectively), there was a general increase in NDVI from April to October. Though sharp declines were observed within months, the highest values were realized in October. This reflects the unimodal rainfall pattern in these provenances. A steep decline in NDVI values was observed from December to March for all the provenances, which are also the driest months of the year. The trends observed in our study strongly agree with the phenology of rosewood populations. Leaf flushing at the start of the rainy season and intense vegetative growth between May and October were also periods of high NDVI measurements indicating peak photosynthetic activity (Duvall, 2008; Pettorelli et al., 2005). The period of fruit maturation (where the samaras turn from green to straw yellow), fruit/seed dispersal, and a subsequent deciduousness of the tree during the dry season between December and March, were the period with low NDVI values indicating reduced photosynthetic activity.

### 3.2 Variation in fruit/seed set, seed morphology, and nutrient composition

**FIGURE 3:**
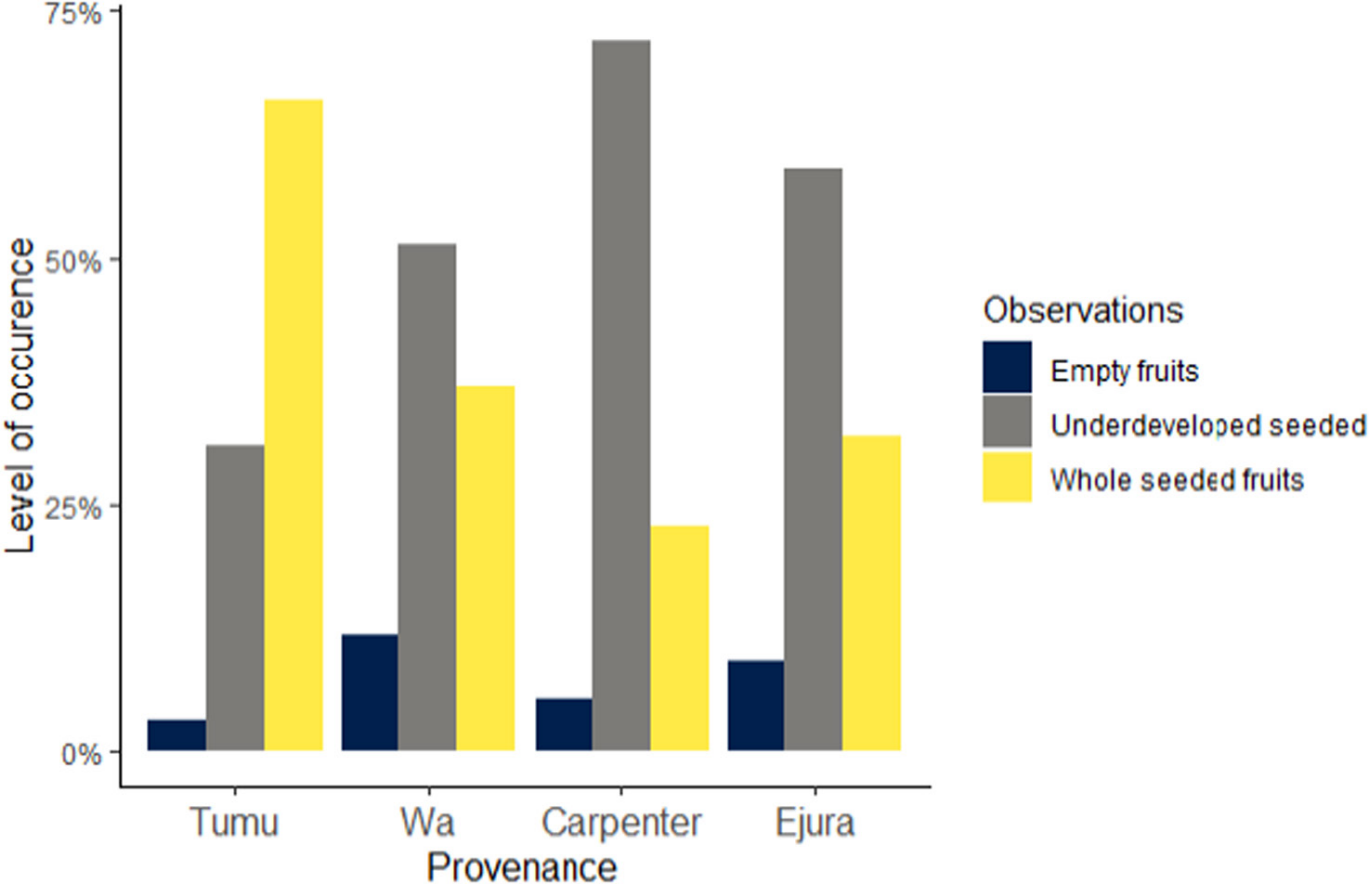
Fruit/Seed set characteristics observed among provenances of *Pterocarpus erinaceus* in Ghana.

Seed set characteristics varied significantly among provenances (Chi‐squared = 147.79, *p*‐value <.001). In Tumu provenance, a greater percentage of fruits contained whole seeds, followed by underdeveloped seeded fruits and empty fruits. The pattern was different in all three remaining provenances where most fruits collected had underdeveloped seeds, followed by whole‐seeded fruits and empty fruits (Figure 3). Seed morphological traits and chemical composition differed significantly among provenances at *p* < .05 and *p* < .01 (Tables 1 and 2, respectively). Seeds from Tumu recorded the highest mass and were followed by seeds from Wa, Carpenter, and Ejura provenances, respectively (Table 1). The mass of seeds from Tumu differed significantly from those of Carpenter and Ejura provenances, but not Wa provenance. Seeds mass from Wa provenance differed significantly from Ejura but not Carpenter provenance. Seed area measurements of the provenances expressed the same pattern as the seed mass. Seed length was similar for all provenances except for Ejura provenance which was significantly smaller than all others.

**TABLE 1:** Differences in seed mass, area, and length among provenances.

Provenance	Seed characteristics		
	Seed mass (g)	Seed area (cm^2^)	Seed length (cm)
Tumu	10.01 ± 0.67*a*	0.73 ± 0.09*a*	1.21 ± 0.08*a*
Wa	7.76 ± 0.*54ab*	0.58 ± 0.*09ab*	1.05 ± 0.10*a*
Carpenter	5.53 ± 0.*36bc*	0.53 ± 0.08*bc*	1.05 ± 0.10*a*
Ejura	4.83 ± 0.18*c*	0.42 ± 0.04*c*	0.90 ± 0.04*b*

*Note*: Values are means ± SD. Seed mass values are means of 8 replicates of 100 seeds each while seed area and length are means of 10 replicates of individual seeds. Same letters in columns represent no significant differences among provenances (*p* < .01 for seed area and *p* < .05 for seed length and mass).

**TABLE 2:** Differences in nutrient composition of seeds among provenances.

Component	(%) Composition			
	Carpenter	Wa	Ejura	Tumu
Moisture content	11.17 ± 0.06*a*	10.82 ± 0.11*ab*	10.75 ± 0.52*ab*	9.80 ± 0.06*b*
Ash	5.01 ± 1.06*a*	5.26 ± 0.14*a*	5.32 ± 0.24*a*	6.17 ± 0.06*a*
Crude fat	15.70 ± 0.62*b*	15.88 ± 0.81*b*	14.03 ± 0.14*b*	18.99 ± 0.66*a*
Crude fiber	11.82 ± 0.01*a*	10.35 ± 0.19*b*	12.13 ± 0.04*a*	9.81 ± 0.03*c*
Crude protein	35.42 ± 0.74*a*	27.52 ± 0.21*c*	30.56 ± 1.01*b*	26.70 ± 0.66*c*
Carbohydrate	20.88 ± 1.01*c*	30.18 ± 0.56*a*	27.21 ± 0.35*b*	28.52 ± 0.51*ab*

*Note*: Values are means ± SD of duplicate determination. Same letters in rows represent no significant differences (*p* < .001, *p* < .01, or *p* < .05) among the study provenances.

The moisture content of seeds ranged between 9.8% and 11.2%, characteristic of seeds with orthodox storage behavior (Table 2). Carbohydrate and crude protein content were relatively high for all provenances, their sum accounting for more than 50% of the total seed chemical content. Crude protein accounted for the highest proportion of nutrient content in seeds of Carpenter and Ejura provenances, whiles carbohydrate was the largest nutrient determined for seeds of Tumu and Wa provenances. Crude fat was the next largest nutrient content of the seeds after crude protein and carbohydrate and was followed by crude fiber and ash contents, respectively. Significant differences in the nutrients among provenances are denoted with letters in Table 2.

### 3.3 Relationship between seed traits and maternal environmental factors

The linear mixed‐effects model including only the variables that collectively improved the model with lower AIC revealed that there were significant relationships between the environmental factors and seed morphological traits. The environmental factors explained between 21% and 66% of variations in the seed morphological traits studied (Table 3). Among the environmental factors, temperature was the strongest predictor of the seed traits showing significant relationships with all the traits studied (Table 3). For instance, when seed mass was analyzed, temperature alone accounted for 21% of the variation observed. For all the seed traits studied, temperature expressed a positive relationship indicating that seed size and mass of African rosewood increased with temperature. On the contrary, absolute rainfall (i.e., total amount of rainfall recorded during the seed production year) expressed a significant and negative relationship with seed length (and hence seed size). Soil nutrients, however, expressed a significant and positive relationship with seed area (Table 3). Soil nitrogen had a strong positive relationship with seed nitrogen (*R* ^2^ = 0.91, *p* < .001).

**TABLE 3:** Effect of environmental factors on seed morphological traits of *Pterocarpus erinaceus.*

Seed trait	Predictor	Estimate	*t*‐value	Pr(>|*t*|)	AIC	*R* ^*2*^ *m*
Seed length	Absolute rainfall	−0.055	−3.389	0.002**	−59.58	0.65
	Mean monthly temperature	0.134	8.241	> 0.001***		
Seed area	1st Soil nutrients axis	0.030	2.209	0.0335*	−62.08	0.66
	Mean monthly temperature	0.119	8.707	> 0.001***		
Seed mass	Mean monthly temperature	0.098	3.213	0.003**	−2.70	0.21

*Note*: Pr(>|*t*|), *p‐*value of *t*‐test; *p < .05; **p < .01; ***p < .001; AIC, Akaike Information Criterion; *R* ^*2*^ *m* indicates marginal *R* ^2^ which is the proportion of variation explained by a model's fixed effects.

### 3.4 Germination response among provenances and the effect of the maternal environment and seed nutrient content

**FIGURE 4:**
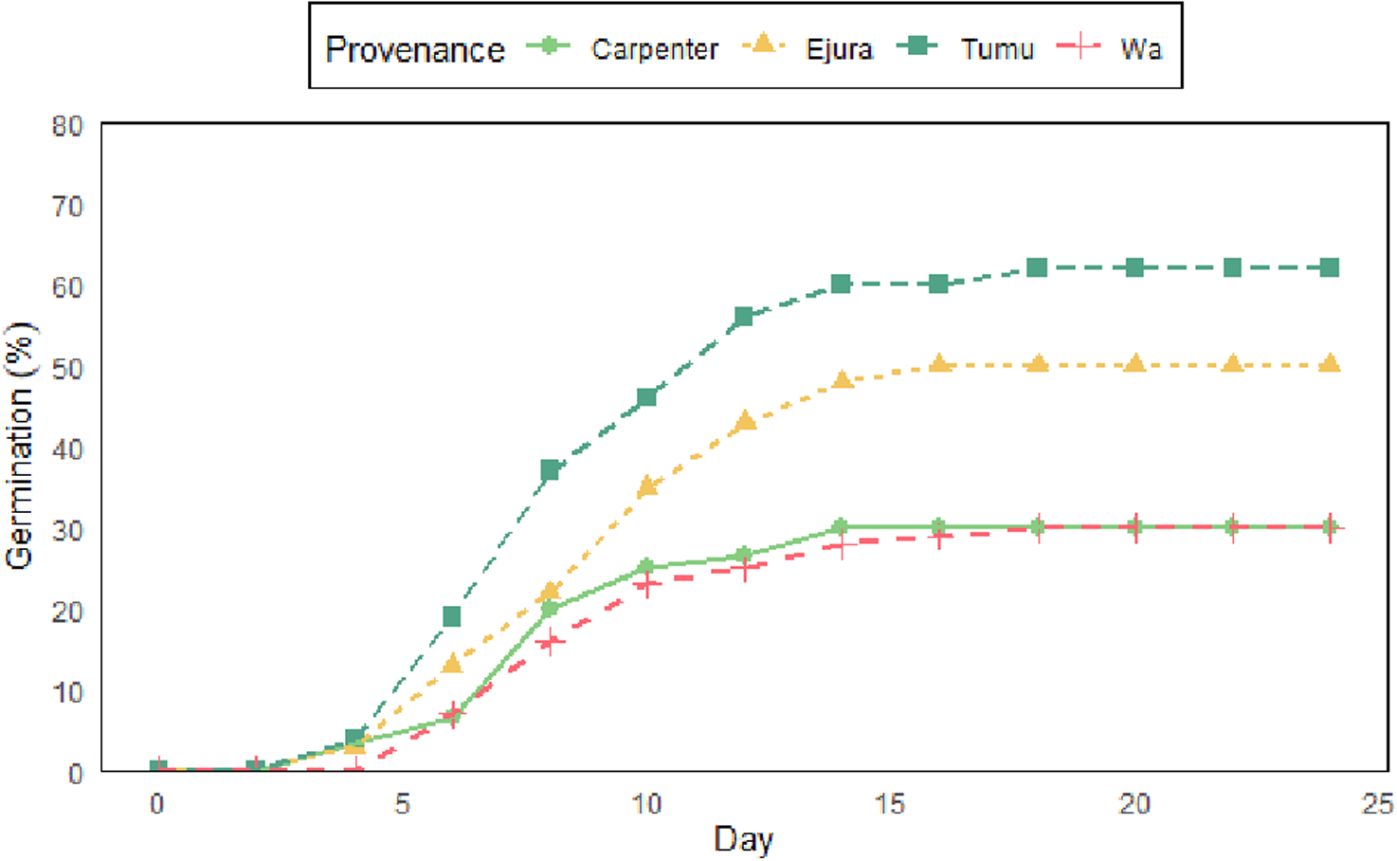
Germination time course of *Pterocarpus erinaceus* seeds from the study provenances.

Mean emergence among provenances was generally low and ranged between 30% (Carpenter and Wa) and 62% (Tumu) (See supplementary Table S3 for detailed germination performance for each replicate batch of seeds per provenance). First time of emergence was 4 days after sowing and peak germination was recorded on the 16th day after sowing for all provenances except for Tumu, where a later emergence was observed on the 18th day after sowing (Figure 4). The germination time course describing the cumulative germination of seeds displayed an S‐shape. The generalized linear model showed no significant association between the maternal environment or the seed nutrient content and the final germination percentages (*p* > .05). However, temperature expressed a positive relationship with the final germination score at a lower likelihood (odds ratio = 1.18, 95% CI, 0.42–3.41), while the soil nutrients showed a negative association with final germination score (odds ratio = 0.82, 95% CI, 0.52–1.23). The odds of germination increased with seed mass (odds ratio = 1.12, 95% CI, 0.71–1.79). Crude protein, carbohydrate, and final germination score were not related (odds ratios = 0.72 and 0.77, 95% CI, 0.28–1.73 and 0.31–1.83, respectively).

### 3.5 Seedling growth performance among provenances

Seedlings from the different provenances expressed similar growth performance at the end of the 6 months observation showing non‐significant differences (*p* > .05) in mean stem length, root length, and root collar diameter (Table 4). Notable variation in root length was observed among seedlings of the same provenances, although they were grown under the same conditions (SD = 15.9 cm for Tumu provenance). Seedlings from Tumu provenance recorded the highest stem length, which was followed by seedlings from Wa and Ejura provenance, respectively. Over the same period, the taproots were twice longer than the stems in all provenances. Wa provenance recorded the highest mean seedlings root length and was followed by Tumu and Ejura provenances, respectively. The highest root collar diameter was obtained for seedlings from Wa provenance and was closely followed by seedlings from Ejura. The mean collar diameter recorded for Tumu was slightly lower, but this difference was not statistically different from the others (*p* > .5).

**TABLE 4:** Seedlings growth measurements among provenances.

Variable	Provenance		
	Wa	Ejura	Tumu
Stem length (cm)	9.41 ± 1.66*a*	9.21 ± 3.86*a*	11.99 ± 2.04*a*
Root collar diameter (mm)	4.79 ± 1.99*a*	4.73 ± 1.19*a*	3.97 ± 0.53*a*
Root length (cm)	28.14 ± 6.66*a*	20.1 ± 5.34*a*	26.10 ± 15.19*a*
RGR_sl_ (cm cm^−1^ week^−1^)	0.017 ± 0.00*a*	0.027 ± 0.01*a*	0.031 ± 0.00*a*
RGR_cd_ (mm mm^−1^ week^−1^)	0.035 ± 0.00*ab*	0.045 ± 0.01*a*	0.029 ± 0.00*b*

*Note*: Mean ± SD in the same row with similar letters have no significant differences (*p* < .05).

Abbreviations: RGR_cd_, relative growth rate of root collar diameter; RGR_sl_, relative growth rate of shoot length.

Seedlings of Tumu provenance recorded the highest relative stem growth rates and were followed by Ejura and Wa provenances, respectively. Significant differences in relative growth rates were only observed in root collar diameter among seedlings of different seed sources. Seedlings from Ejura provenance recorded the highest root collar diameter growth rates and were statistically different from seedlings of Tumu provenance, but did not differ from seedlings of Wa provenance (Table 4).

## 4 DISCUSSION

### 4.1 Patterns of seed characteristics among provenances

Substantial variations in African rosewood seed traits were observed among the studied provenances. Seed size and mass increased with increasing distance of the seed sources from the forest‐savanna transition toward the Guineo‐Sudanian savanna regions (Figure 1). Intraspecific variations in seed size and mass along geographical and environmental gradients have been reported in several studies (Carón et al., 2014; Gorden et al., 2016; Wu et al., 2018). The considerable differences in size and mass of African rosewood seeds among provenances may be explained by differences in climatic factors, soil nutrients, and levels of environmental stress that occur in the different locations. For instance, bigger seed mass and size have been reported to improve germination success and early seedling establishment, thus, it is common for mother plants growing in stressed environments to produce bigger seeds even if that implies reducing the number of seeds they produce (Wu et al., 2018). This is possible because in many instances, seed dispersal is often limited and hence offspring grow in a similar environment as their maternal plant (Galloway & Etterson, 2007). Bigger seed mass has also been linked to several important plant functions such as maintenance or increase in biomass, defense mechanisms, and transfer of hereditary materials to offspring (Redmond et al., 2019). Hence, it is not surprising that for provenances in high‐temperature environments, mother plants provisioned seeds more in mass and size to improve survival and fitness against pronounced stress conditions, consistent with our first hypothesis.

The occurrence of underdeveloped seeds and parthenocarpic samara in *Pterocarpus erinaceus* was reported in a recent study in Ghana (Tiika et al., 2019). In that study, the phenomenon reduced the chances of whole and unblemished seeds (possibly viable seeds) to less than 50% of the total seed set. Their findings are consistent with the results of this study but more importantly, we note that variations exist among provenances. This phenomenon can be very disturbing for commercial seed collectors and nursery operators and with a high reproduction cost to the plants. The phenomenon of underdeveloped seeds in matured and dispersing samaras of African rosewood remains unexplained but might arise as a result of pre‐dispersal seed predation during seed set or the occurrence of late seed set (Perea et al., 2013). Nonetheless, this can confer an advantage to the fruiting trees (Traveset, 1993; Zangerl et al., 1991). For instance, Perea et al. (2013) reported that the increasing occurrence of empty samaras in *Ulmus laevis* was associated with a decrease in pre‐dispersal seed predation rate. They explain that the phenomenon may increase the number of viable seeds that escape predation. While this could be a possible explanation for the seed set observations in African rosewood, we advocate for further research to arrive at a definitive conclusion.

Crude protein and carbohydrate contents were found consistently high for all provenances. This implies that seeds of African rosewood are naturally rich sources of carbohydrate and protein nutrients. Carpenter and Ejura provenances with the highest seed crude protein contents were also associated with the highest soil organic matter and total nitrogen contents. It is suggested that seed nutrient composition could vary with the amount and availability of nutrients to mother plants (Carón et al., 2014; De Frenne et al., 2011). The low‐seed shedding moisture of less than 12% obtained for all provenances is characteristic of seeds with orthodox storage behavior (Asomaning, 2018). Such seeds undergo maturation drying and can remain viable in ex‐situ storage over extended periods (Amponsah et al., 2022).

### 4.2 Effects of the maternal environment on seed traits

We predicted that maternal environments (provenances) with limited resources and/or with pronounced environmental stress would influence the adjustment of seed morphological traits to have a better competitive advantage (i.e., larger sizes and bigger mass). Our findings are partially in support of this hypothesis as maternal environmental factors strongly influenced seed traits in different ways. Temperature and soil nutrients showed strong significant positive relationships with seed size traits (i.e., seed area and/or seed length), whereas absolute rainfall expressed a significant negative relationship with seed length.

Maternal environmental factors including temperature, rainfall, and solar radiation are known to have important effects on seed traits, being attributed to account for the underlying mechanisms in geographical gradients of seed size (Murray et al., 2004). While high temperatures may pose environmental stress to plants, they also favor the rate of photosynthesis and hence the amount of photosynthates produced, which possibly could result in an increased allocation of biomass to seeds (Wu et al., 2018). This may explain the strong significant positive relationships between seed size traits and temperature in African rosewood. An alternative explanation is that, at high‐temperature environments, the cost of metabolism for seed germination and plant growth is greater, so that producing larger seeds is necessary to support larger vigorous well‐resourced seedlings that can cope (Lord et al., 1997; Murray et al., 2004).

The provenances studied had similar seasonality indexes indicating like patterns in rainfall distribution. Consequently, absolute rainfall amounts over the seed production year were similar among provenances. However, we found that high rainfall amounts in a maternal environment were associated with reduced seed sizes hence reducing the competitive capacities of seeds. This observation may partly account for the wide distribution of the species in the dry savannah zones, and its notable absence in the moist and wet ecological zones (Duvall, 2008).

Soil nutrients of the maternal environment expressed a significant positive association with seed size (seed area). Again, seed nitrogen content strongly correlated with soil nitrogen content in agreement with hypothesis 2a. Our results are consistent with the findings of other studies that have reported a relationship between soil nutrients and seed traits including nutrient composition (Carón et al., 2014; De Frenne et al., 2011; Galloway, 2001; Wu et al., 2018). For instance, soil carbon consistently related positively to seed carbon content in *Anemone nemoras*, while seed N:P decreased with increasing available soil nutrients (De Frenne et al., 2011). Furthermore, seed nutrient content was closely related to temperature in our study and expressed similar effects, accounting for relative variance explained from the models. This result is consistent with several others (De Frenne et al., 2011; Wu et al., 2018) which conclude that the relationship between seed nutrients, size, and soil nutrients may result from increased seed provisioning due to increased soil nutrient availability.

### 4.3 Germination capacity among provenances and its relationship with seed traits

Seeds from Tumu and Ejura provenances attained 50% germination and more, while seeds from Wa and Carpenter provenances recorded very low emergence of just about 30% final germination. The relatively low germination observed in our study, coupled with concurring high occurrence of underdeveloped seeds suggests the possible destruction of seed embryo by insect larvae, which renders most seeds unviable. Again, this is likely considering that African rosewood seeds are not associated with deep dormancy, and some studies suggest that the species do not require dormancy‐breaking treatments prior to sowing (Zida et al., 2005). Other studies have also reported low germination (Akpona et al., 2017, several unpublished student theses and germination records at the NTSC), but see Amponsah et al. (2022), Duvall (2008), Tiika et al. (2019) and Zida et al. (2005). Our results, however, show that significant differences exist among provenances.

Consistent with our initial hypothesis, a bigger seed mass improved germination outcome. The relationship between seed mass and germination success has been reported in several studies as well (e.g., Baskin & Baskin, 2014; Moles & Westoby, 2006; Wu et al., 2018). The positive association between seed mass and germination outcome may be explained by the higher amount of nutrient reserves in seeds of bigger mass, which supply energy to the germinating embryo and for the successful establishment of seedlings (De Frenne et al., 2011; Pérez‐Ramos et al., 2010).

### 4.4 Seedling growth among provenances

At the end of the growth experiment, regardless of their seed source or sizes, the seedlings reached similar growth rates and sizes. This was in disagreement with our third hypothesis, where we expected seedlings from large seeds to be of bigger sizes. The 6‐month‐long duration of the experiment coupled with a common growth environment for all the seedlings recruited in this study may account for the similarity in their growth rates and sizes (Zida et al., 2008). While seedlings of larger seeds generally start out bigger, this initial advantage persists for a given amount of time, which depends on the species (Moles & Westoby, 2006). It is, therefore, likely that seed mass effect on seedlings size in our experiment might have occurred at an earlier stage in the seedlings growth. Again, this is possible considering that seed mass significantly influenced germination performance and seedling emergence. Hence, while seedlings from bigger seeds might have launched out bigger, the initial advantage may have been canceled out over the 6‐months long duration. Seedlings from Ejura recorded the highest significant growth rates over the experimental period. The relatively high crude protein content in the seeds from Ejura provenance might explain this observation, which could have promoted growth rates and compensated for the smaller sizes of the seeds. The significance of seed nutrient concentration in successful seedling establishment and growth has been documented in other studies (e.g., Carón et al., 2014; Pérez‐Ramos et al., 2010).

## 5 CONCLUSION

We found significant variations in the seed size and mass traits of African rosewood, proportion of fruits that contained whole seeds, and germination capacity among provenances, which were related to maternal environmental factors. The results indicate that temperature is the key environmental factor that defines seed traits among populations of the species in Ghana. These findings point to the importance of provenances in maximizing the cultivation success of the species. In particular, we suggest prioritizing provenances in the Tumu and Upper West regions of the country for propagation and plant regeneration. Furthermore, considering the high levels of seed predation prior to dispersal among the provenances which reduced the viability of seeds, we recommend future studies to ascertain the prevalence of this phenomenon and its impact on populations of the species. There is also a need for developing technologies that improve the growth rate of the species to meet local demands and its utilization in plantation programs. Finally, our results highlight the adaptive strategies by mother plants to enhance offspring fitness in African rosewood. The study therefore makes an important contribution to restoring the populations of this endangered species.

## ACKNOWLEDGEMENTS

Special thanks to the National Tree Seed Centre, CSIR‐Forestry Research Institute of Ghana and Asare‐Gyebi Kwadwo for the assistance with seed collection. Thanks to the three anonymous reviewers for their helpful comments on the earlier version of this manuscript.

## FUNDING INFORMATION

This research did not receive any specific grant from funding agencies in the public, commercial, or not‐for‐profit sectors.

## CONFLICT OF INTEREST STATEMENT

The authors declare no conflict of interest.

## Supporting information


Data S1.

## Data Availability

The data associated with this study would be archived at Dryad upon acceptance.

## References

[pei310120-bib-0001] Adjonou, K. , Abotsi, K. E. , Segla, K. N. , Rabiou, H. , Houetchegnon, T. , Sourou, K. B. , Johnson, B. N. , Ouinsavi, C. A. N. , Kokutse, A. D. , Mahamane, A. , & Kokou, K. (2020). Vulnerability of African rosewood (*Pterocarpus erinaceus*, Fabaceae) natural stands to climate change and implications for silviculture in West Africa. Heliyon, 6(6), e04031. 32518851 10.1016/j.heliyon.2020.e04031 PMC7270547

[pei310120-bib-0002] Akpona, T. J. D. , Amagnide, A. G. , Assogbadjo, A. , & Kakaï, R. G. (2017). Factors affecting *Pterocarpus erinaceus* Poir. seed germination and seedlings growth in the Republic of Benin. Annales Des Sciences Agronomiques, 21(2), 167–180.

[pei310120-bib-0003] Amissah, L. , Mohren, G. M. , Kyereh, B. , & Poorter, L. (2015). The effects of drought and shade on the performance, morphology and physiology of Ghanaian tree species. PLoS One, 10(4), e0121004. 25836337 10.1371/journal.pone.0121004 PMC4383566

[pei310120-bib-0004] Amponsah, J. O. , Asomaning, J. M. , Gakpetor, P. M. , & Gaveh, E. A. (2022). Seed germination, storability and moisture sorption isotherms of the endangered African rosewood (Pterocarpus erinaceus). Journal of Horticulture and Forestry, 14(1), 1–9.

[pei310120-bib-0005] Arbonnier, M. (2002). Trees, shrubs and lianas of the dry areas of West Africa. CIRAD Editions.

[pei310120-bib-0006] Asomaning, J. M. (2018). A guidebook for tree seed testing (p. 59). CSIR‐Forestry Research Institute of Ghana.

[pei310120-bib-0007] Association of Official Analytical Chemist . (2002). Official method of analysis of the Association of Official Analytical Chemist (14th ed.). Association of Official Analytical Chemists.

[pei310120-bib-0008] Barstow, M. (2018). Pterocarpus erinaceus. The IUCN Red List of Threatened Species 2018: e.T62027797A62027800. 10.2305/IUCN.UK.2018-2.RLTS.T62027797A62027800.en

[pei310120-bib-0009] Baskin, C. C. , & Baskin, J. M. (2014). Seeds: Ecology, biogeography, and evolution of dormancy and germination. Elsevier Ltd.

[pei310120-bib-0010] Bradford, K. , & Nonogaki, H. (2008). Annual plant reviews, seed development, dormancy and germination. Wiley‐Blackwell.

[pei310120-bib-0011] Calvo, L. , Hernández, V. , Valbuena, L. , & Taboada, A. (2016). Provenance and seed mass determine seed tolerance to high temperatures associated to forest fires in *Pinus pinaster* . Annals of Forest Science, 73(2), 381–391.

[pei310120-bib-0012] Carón, M. M. , De Frenne, P. , Brunet, J. , Chabrerie, O. , Cousins, S. A. , De Backer, L. , Diekmann, M. , Graae, B. J. , Heinken, T. , Kolb, A. , & Naaf, T. (2014). Latitudinal variation in seeds characteristics of *Acer platanoides* and *A. pseudoplatanus* . Plant Ecology, 215(8), 911–925.

[pei310120-bib-0013] Cochrane, A. , Yates, C. J. , Hoyle, G. L. , & Nicotra, A. B. (2015). Will among‐population variation in seed traits improve the chance of species persistence under climate change? Global Ecology and Biogeography, 24(1), 12–24.

[pei310120-bib-0014] De Frenne, P. , Kolb, A. , Graae, B. J. , Decocq, G. , Baltora, S. , De Schrijver, A. , Brunet, J. , Chabrerie, O. , Cousins, S. A. , Dhondt, R. , & Diekmann, M. (2011). A latitudinal gradient in seed nutrients of the forest herb *Anemone nemorosa* . Plant Biology, 13(3), 493–501. 21489100 10.1111/j.1438-8677.2010.00404.x

[pei310120-bib-0015] Donohue, K. (2009). Completing the cycle: Maternal effects as the missing link in plant life histories. Philosophical Transactions of the Royal Society B: Biological Sciences, 364(1520), 1059–1074. 10.1098/rstb.2008.0291 PMC2666684 19324611

[pei310120-bib-0016] Dumenu, W. K. (2019). Assessing the impact of felling/export ban and CITES designation on exploitation of African rosewood (*Pterocarpus erinaceus*). Biological Conservation, 236, 124–133.

[pei310120-bib-0017] Duvall, C. S. (2008). In D. Louppe , A. A. Oteng‐Amoako , & M. Brink (Eds.), Pterocarpus erinaceus Poir. PROTA (Plant Resources of Tropical Africa / Ressources végétales de l'Afrique tropicale).

[pei310120-bib-0018] Fenner, M. , & Thompson, K. (2005). The ecology of seeds. Cambridge University Press.

[pei310120-bib-0019] Galloway, L. F. (2001). The effect of maternal and paternal environments on seed characters in the herbaceous plant *Campanula Americana* (Campanulaceae). American Journal of Botany, 88(5), 832–840. 11353708

[pei310120-bib-0020] Galloway, L. F. (2005). Maternal effects provide phenotypic adaptation to local environmental conditions. New Phytologist, 166(1), 93–100. 15760354 10.1111/j.1469-8137.2004.01314.x

[pei310120-bib-0021] Galloway, L. F. , & Etterson, J. R. (2007). Transgenerational plasticity is adaptive in the wild. Science, 318(5853), 1134–1136. 18006745 10.1126/science.1148766

[pei310120-bib-0022] Gianinetti, A. (2020). Basic features of the analysis of germination data with generalized linear mixed models. Data, 5(1), 6.

[pei310120-bib-0023] Gorden, N. L. S. , Winkler, K. J. , Jahnke, M. R. , Marshall, E. , Horky, J. , Hudelson, C. , & Etterson, J. R. (2016). Geographic patterns of seed mass are associated with climate factors, but relationships vary between species. American Journal of Botany, 103(1), 60–72. 26758888 10.3732/ajb.1500295

[pei310120-bib-0024] Gorelick, N. , Hancher, M. , Dixon, M. , Ilyushchenko, S. , Thau, D. , & Moore, R. (2017). Google Earth Engine: Planetary‐scale geospatial analysis for everyone. Remote Sensing of Environment, 202, 18–27.

[pei310120-bib-0025] Hachem, S. , Duguay, C. R. , & Allard, M. (2012). Comparison of MODIS‐derived land surface temperatures with ground surface and air temperature measurements in continuous permafrost terrain. The Cryosphere, 6(1), 51–69.

[pei310120-bib-0026] Herman, J. J. , & Sultan, S. E. (2011). Adaptive transgenerational plasticity in plants: Case studies, mechanisms, and implications for natural populations. Frontiers in Plant Science, 2, 102. 22639624 10.3389/fpls.2011.00102 PMC3355592

[pei310120-bib-0027] Hoffmann, W. A. , & Poorter, H. (2002). Avoiding bias in calculations of relative growth rate. Annals of Botany, 90(1), 37–42. 12125771 10.1093/aob/mcf140 PMC4233846

[pei310120-bib-0028] Jo, C. , & Wilson, E. R. (2005). The importance of plus‐tree selection in the improvement of hardwoods. Quarterly Journal of Forestry, 99, 45–50.

[pei310120-bib-0029] Korang, J. K. , Obiri, B. D. , Appiah, H. , & Awuku, S. (2015). Calorific values and gravimetric yield of six wood fuel species in the forest transition zone of Ghana. Ghana Journal of Forestry, 31, 51–61.

[pei310120-bib-0030] Kyei, K. R. (2016). Effect of different pre‐sowing treatments on the germination and initial growth of Pterocarpus erinaceus seeds. Masters Dissertation, (p. 70). Kwame Nkrumah University of Science and Technology—Kumasi.

[pei310120-bib-0031] Lawson, S. (2015). The illegal rosewood boom in West Africa; How Chinese demand is driving conflict, corruption and human rights abuses. In *Presentation to Chatham House Illegal Logging Stakeholder Update Meeting*, *25th* June.

[pei310120-bib-0032] Livada, I. , & Asimakopoulos, D. N. (2005). Individual seasonality index of rainfall regimes in Greece. Climate Research, 28(2), 155–161.

[pei310120-bib-0033] Lord, J. , Egan, J. , Clifford, T. , Jurado, E. , Leishman, M. , Williams, D. , & Westoby, M. (1997). Larger seeds in tropical floras: Consistent patterns independent of growth form and dispersal mode. Journal of Biogeography, 24(2), 205–211.

[pei310120-bib-0035] McCarthy, B. C. (1997). Lab protocols for the testing of eastern deciduous forest soils. Department of Environmental and Plant Biology, Ohio University.

[pei310120-bib-0036] Meneses‐Tovar, C. L. (2011). NDVI as indicator of degradation. Unasylva, 62(238), 39–46.

[pei310120-bib-0037] Messier, J. , McGill, B. J. , & Lechowicz, M. J. (2010). How do traits vary across ecological scales? A case for trait‐based ecology. Ecology Letters, 13(7), 838–848. 20482582 10.1111/j.1461-0248.2010.01476.x

[pei310120-bib-0038] Mkhabela, M. S. , Bullock, P. , Raj, S. , Wang, S. , & Yang, Y. (2011). Crop yield forecasting on the Canadian Prairies using MODIS NDVI data. Agricultural and Forest Meteorology, 151(3), 385–393.

[pei310120-bib-0040] Moles, A. T. , & Westoby, M. (2006). Seed size and plant strategy across the whole life cycle. Oikos, 113(1), 91–105.

[pei310120-bib-0041] Murray, B. R. , Brown, A. H. D. , Dickman, C. R. , & Crowther, M. S. (2004). Geographical gradients in seed mass in relation to climate. Journal of Biogeography, 31(3), 379–388.

[pei310120-bib-0042] Ouédraogo, A. , Thiombiano, A. , Hahn‐Hadjali, K. , & Guinko, S. (2006). Diagnostic de l'état de dégradation des peuplements de quatre espèces ligneuses en zone soudanienne du Burkina Faso. Science et Changements Planétaires/Sécheresse, 17(4), 485–491.

[pei310120-bib-0043] Penfield, S. , & MacGregor, D. R. (2017). Effects of environmental variation during seed production on seed dormancy and germination. Journal of Experimental Botany, 68(4), 819–825. 27940467 10.1093/jxb/erw436

[pei310120-bib-0044] Perea, R. , Venturas, M. , & Gil, L. (2013). Empty seeds are not always bad: Simultaneous effect of seed emptiness and masting on animal seed predation. PLoS One, 8(6), e65573. 23776503 10.1371/journal.pone.0065573 PMC3679161

[pei310120-bib-0045] Pérez‐Ramos, I. M. , Gómez‐Aparicio, L. , Villar, R. , García, L. V. , & Maranon, T. (2010). Seedling growth and morphology of three oak species along field resource gradients and seed mass variation: A seedling age‐dependent response. Journal of Vegetation Science, 21(3), 419–437.

[pei310120-bib-0046] Pettorelli, N. , Vik, J. O. , Mysterud, A. , Gaillard, J. M. , Tucker, C. J. , & Stenseth, N. C. (2005). Using the satellite‐derived NDVI to assess ecological responses to environmental change. Trends in Ecology & Evolution, 20(9), 503–510. 16701427 10.1016/j.tree.2005.05.011

[pei310120-bib-0047] Poorter, L. (1999). Growth responses of 15 rain‐forest tree species to a light gradient: The relative importance of morphological and physiological traits. Functional Ecology, 13(3), 396–410.

[pei310120-bib-0048] R. Development Core Team . (2015). R: A language and environment for statistical computing. R Foundation For Statistical Computing.

[pei310120-bib-0049] Redmond, M. D. , Davis, T. S. , Ferrenberg, S. , & Wion, A. P. (2019). Resource allocation trade‐offs in a mast‐seeding conifer: piñon pine prioritizes reproduction over defence. AoB Plants, 11(6), plz070.

[pei310120-bib-0050] Royal Botanic Gardens, Kew . (2005). A field manual for seed collectors. Wakehurst Place.

[pei310120-bib-0051] Schneider, C. A. , Rasband, W. S. , & Eliceiri, K. W. (2012). NIH Image to ImageJ: 25 years of image analysis. Nature Methods, 9, 671–675. 22930834 10.1038/nmeth.2089 PMC5554542

[pei310120-bib-0052] Segla, N. K. , Rabiou, H. , Adjonou, K. , Moussa, B. M. , Saley, K. , Radji, R. A. , Kokutse, A. D. , Bationo, A. B. , Mahamane, A. , & Kokou, K. (2016). Population structure and minimum felling diameter of *Pterocarpus erinaceus* Poir in arid and semi‐arid climate zones of West Africa. South African Journal of Botany, 103, 17–24.

[pei310120-bib-0053] TeKrony, D. M. , & Hardin, E. E. (1969). The problem of underdeveloped seeds occurring in monogerm sugarbeets. Journal of the American Society of Sugar Beet Technologists, 15, 625–639.

[pei310120-bib-0054] Tiika, R. J. , Issifu, H. , Baatuuwie, B. N. , Nasare, L. I. , & Husseini, R. (2019). Seed quality, germinability and initial growth of *Pterocarpus erinaceus* (African rosewood). How important are mother tree size, source and timing of fruit harvest? Journal of Forest and Environmental Science, 35(2), 69–77.

[pei310120-bib-0055] Traveset, A. (1993). Deceptive fruits reduce seed predation by insects in *Pistacia terebinthus L*. (Anacardiaceae). Evolutionary Ecology, 7(4), 357–361.

[pei310120-bib-0056] Walsh, R. P. D. , & Lawler, D. M. (1981). Rainfall seasonality: Description, spatial patterns and change through time. Weather, 36(7), 201–208.

[pei310120-bib-0057] Wu, H. , Meng, H. , Wang, S. , Wei, X. , & Jiang, M. (2018). Geographic patterns and environmental drivers of seed traits of a relict tree species. Forest Ecology and Management, 422, 59–68.

[pei310120-bib-0058] Zangerl, A. R. , Berenbaum, M. R. , & Nitao, J. K. (1991). Parthenocarpic fruits in wild parsnip: Decoy defence against a specialist herbivore. Evolutionary Ecology, 5(2), 136–145.

[pei310120-bib-0059] Zas, R. , Cendán, C. , & Sampedro, L. (2013). Mediation of seed provisioning in the transmission of environmental maternal effects in maritime pine (*Pinus pinaster* Aiton). Heredity, 111(3), 248–255. 23652562 10.1038/hdy.2013.44 PMC3746824

[pei310120-bib-0060] Zida, D. , Tigabu, M. , Sawadogo, L. , & Oden, P. C. (2005). Germination requirements of seeds of four woody species from the Sudanian savanna in Burkina Faso, West Africa. Seed Science and Technology, 33(3), 581–593.

[pei310120-bib-0061] Zida, D. , Tigabu, M. , Sawadogo, L. , & Odén, P. C. (2008). Initial seedling morphological characteristics and field performance of two Sudanian savanna species in relation to nursery production period and watering regimes. Forest Ecology and Management, 255(7), 2151–2162.

