# Supplementary material for: Temperature and soil nutrients drive seed traits variation in Pterocarpus erinaceus (African rosewood) in Ghana
Source: Plant Environ Interact. 2023 Jul 25;4(4):215–27. doi: 10.1002/pei3.10120 (PMC10423977; doi:10.1002/pei3.10120)
Supplement: Supplementary file 1 — Data S1. [file PEI3-4-215-s001.docx]

**Supplementary materials**

**Supplementary Figure 1**
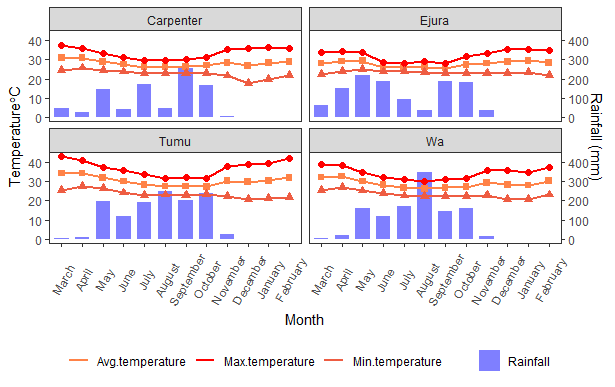


Supplementary Figure 1. Climograph of the study provenances during seed production year

**Supplementary Table 1. Soil physicochemical characteristics in the four provenances**

|  | Ejura | Wa | Carpenter | Tumu |
| --- | --- | --- | --- | --- |
| pH | 6.84±0.01*a* | 6.1±0.14*b* | 5.50±0.14*c* | 5.53±0.01*c* |
| % O.C | 1.39±0.03*b* | 1.24+0.01*c* | 2.41±0.02*a* | 0.88±0.02*d* |
| % T. N | 0.14±0.01*b* | 0.13±0.01*b* | 0.24±0.01*a* | 0.08±0.00*c* |
| % O.M | 2.38±0.04*b* | 2.11±0.01*c* | 4.14±0.03*a* | 1.48±0.01d |
| Ca me/100g | 3.67±0.07*a* | 2.99±0.01*b* | 3.65±0.04*a* | 2.99±0.01*b* |
| Mg me/100g | 0.95±0.02*d* | 4.46±0.01*a* | 2.76±0.02*b* | 1.82±0.01*c* |
| K me/100g | 0.15±0.01*c* | 0.27±0.01*b* | 0.26±0.01*b* | 0.42±0.02*a* |
| Na me/100g | 0.08±0.00*ab* | 0.09±0.00*a* | 0.07±0.00*b* | 0.06±0.01*c* |
| T.E.B me/100g | 4.82±0.02*c* | 7.6±0.28*a* | 6.73±0.03*b* | 5.31±0.01*c* |
| Ex.Acidity | 0.12±0.01*c* | 0.16±0.01*c* | 0.61±0.01*b* | 0.86±0.01*a* |
| E.C.E.C me/100g | 4.91±0.01*d* | 7.93±0.03*a* | 7.34±0.02*b* | 6.14±0.02*c* |
| % B.S | 97.93±0.05*a* | 98.05±0.08*a* | 91.82±0.04*b* | 86.20±0.03*c* |
| ppm P | 39.64±0.02*a* | 11.87±0.06*c* | 9.36±0.69*d* | 22.30±0.03*b* |
| % SAND | 92.50±0.71*a* | 71.00±1.41*b* | 57.00±1.41*c* | 93.50±0.71*a* |
| % SILT | 4.00±0.00*c* | 17.00±0.00*b* | 33.00±1.41*a* | 4.00±0.00*c* |
| % CLAY | 3.50±0.71*b* | 12.00±1.41*a* | 10.00±0.00*a* | 2.50±0.71*b* |

Values represent means +standard deviations. O.C. is organic carbon, T.N. is total nitrogen, O. M. is organic matter, Ca is calcium, Mg is magnesium, K is potassium, Na is sodium, T.E.B is total exchangeable bases, Ex.Acidity is exchangeable acidity, E.C.E.C is effective cation exchange capacity, B.S is base saturation and P is phosphorus.

**Supplementary Table 2: Principal component analysis of the soil characteristics**

Importance of components:

PC1 PC2 PC3 PC4 PC5 PC6

Standard deviation 2.747 2.220 1.854 0.213 0.172 0.102

Proportion of Variance 0.472 0.308 0.215 0.003 0.002 0.001

Cumulative Proportion 0.472 0.779 0.994 0.997 0.999 1

PC1 PC2 PC3

pH -0.39463941 -0.874816969 0.26227153

X...O.C 0.73948547 -0.261625449 -0.62009336

X...T.N 0.75810641 -0.303726891 -0.56468500

X...O.M 0.73644046 -0.264908287 -0.62189961

Ca.me.100g 0.13125892 -0.663130152 -0.73526707

Mg.me.100g 0.74710847 0.212728384 0.62795901

K.me.100g -0.10806141 0.987903236 0.08343336

Na.me.100g 0.38550033 -0.710407039 0.57105706

T.E.B.me.100g 0.87353462 0.145140799 0.46015645

Ex.Acidity -0.08203332 0.874456257 -0.47233499

E.C.E.C..me.100g 0.84944920 0.369466739 0.37518129

X...B.S 0.22279642 -0.86969585 0.43406927

ppm.P -0.84067120 -0.526651711 -0.12233515

X..SAND -0.98899189 -0.00867457 0.13774563

X..SILT 0.95159084 0.042818645 -0.29860910

**Supplementary Table 3: Summary of germination performance of replicate batches of seeds per provenance**

| Provenance | Rep | Seeds | grs | grp | mgt | mgr | gsp | unc | syn | vgt | sdg | cvg |
| --- | --- | --- | --- | --- | --- | --- | --- | --- | --- | --- | --- | --- |
| Tumu | 1 | 20 | 10 | 50 | 8.40 | 0.12 | 11.90 | 2.17 | 0.16 | 21.56 | 4.64 | 55.27 |
| Tumu | 2 | 20 | 12 | 60 | 9.33 | 0.11 | 10.71 | 2.19 | 0.17 | 26.73 | 5.17 | 55.39 |
| Tumu | 3 | 20 | 17 | 85 | 9.18 | 0.11 | 10.90 | 2.63 | 0.13 | 26.25 | 5.12 | 55.83 |
| Tumu | 4 | 20 | 10 | 50 | 9.20 | 0.11 | 10.87 | 1.85 | 0.22 | 25.33 | 5.03 | 54.71 |
| Tumu | 5 | 20 | 13 | 65 | 8.31 | 0.12 | 12.04 | 2.29 | 0.18 | 20.50 | 4.53 | 54.50 |
| Wa | 1 | 20 | 5 | 25 | 7.60 | 0.13 | 13.16 | 1.52 | 0.20 | 18.75 | 4.33 | 56.98 |
| Wa | 2 | 20 | 4 | 20 | 8.00 | 0.13 | 12.50 | 1.00 | 0.33 | 22.67 | 4.76 | 59.51 |
| Wa | 3 | 20 | 5 | 25 | 8.40 | 0.12 | 11.90 | 1.37 | 0.30 | 23.25 | 4.82 | 57.40 |
| Wa | 4 | 20 | 9 | 45 | 10.67 | 0.09 | 9.38 | 2.20 | 0.14 | 35.75 | 5.98 | 56.05 |
| Wa | 5 | 20 | 7 | 35 | 10.86 | 0.09 | 9.21 | 1.84 | 0.19 | 37.33 | 6.11 | 56.28 |
| Carpenter | 1 | 20 | 7 | 35 | 7.71 | 0.13 | 12.96 | 1.38 | 0.33 | 20.17 | 4.49 | 58.21 |
| Carpenter | 2 | 20 | 6 | 30 | 10.33 | 0.10 | 9.68 | 1.92 | 0.13 | 33.40 | 5.78 | 55.93 |
| Carpenter | 3 | 20 | 5 | 25 | 7.60 | 0.13 | 13.16 | 1.52 | 0.20 | 18.75 | 4.33 | 56.98 |
| Ejura | 1 | 20 | 8 | 40 | 9.75 | 0.10 | 10.26 | 1.91 | 0.18 | 29.86 | 5.46 | 56.04 |
| Ejura | 2 | 20 | 12 | 60 | 11.00 | 0.09 | 9.09 | 2.19 | 0.17 | 35.27 | 5.94 | 53.99 |
| Ejura | 3 | 20 | 13 | 65 | 9.08 | 0.11 | 11.02 | 2.66 | 0.10 | 25.92 | 5.09 | 56.09 |
| Ejura | 4 | 20 | 9 | 45 | 8.22 | 0.12 | 12.16 | 1.89 | 0.19 | 20.13 | 4.49 | 54.56 |
| Ejura | 5 | 20 | 8 | 40 | 8.75 | 0.11 | 11.43 | 1.91 | 0.18 | 23.29 | 4.83 | 55.15 |

Where rep is replicate, grs is number of germinated seeds, grp is germination percent (%), mgt is mean germination time (days), mgr is mean germination rate (day^-1^), ucn is uncertainty index (bit), syn is synchronization index, vgt is germination variance (day^2^), sdg is germination standard deviation (day), and cvg is coefficient of variation (%).
